# From development to dissection: A coaching‐focused workshop for anatomy educators from the viewpoint of the coaches

**DOI:** 10.1002/ase.70323

**Published:** 2026-07-31

**Authors:** Danielle N. Edwards, Bobbie J. Leeper, Kelsey E. Stevens, Jonathan J. Wisco, Abbey Breckling, Rhiannon E. Robinson, Jeremy J. Grachan

**Affiliations:** ^1^ Department of Cell, Developmental, and Integrative Biology University of Alabama at Birmingham Heersink School of Medicine Birmingham Alabama USA; ^2^ Biology and Physician Assistant Programs Seton Hill University Greensburg Pennsylvania USA; ^3^ Exercise and Health Sciences Department Briar Cliff University Sioux City Iowa USA; ^4^ Department of Anatomy and Neurobiology Boston University Aram V. Chobanian & Edward Avedisian School of Medicine Boston Massachusetts USA; ^5^ Department of Anatomy and Cell Biology College of Medicine, University of Illinois at Chicago Chicago Illinois USA; ^6^ Department of Medicine Rutgers New Jersey Medical School Newark New Jersey USA; ^7^ Present address: University of Louisville, School of Medicine Louisville Kentucky USA

**Keywords:** coaching, continuing education, dissection, workshop

## Abstract

The use of different coaching models can provide an effective environment for skill learning. The Anatomical Donor Stewardship (ADS) Coaching subcommittee, within the Human Anatomy and Physiology Society (HAPS), developed and held an inaugural Coaching workshop for human anatomical dissection for HAPS members at the 2025 HAPS Annual Conference in Pittsburgh, Pennsylvania. This article describes the workshop, as well as impact statements from the coaches. Based on the reception of this model, future coaching workshops should be developed to meet the needs of anatomists at all levels, with support from professional societies.

## INTRODUCTION

### Overview of the Human Anatomy and Physiology Society and their Anatomical Donor Stewardship Committee

The Human Anatomy and Physiology Society's (HAPS) Anatomical Donor Stewardship (ADS) Committee serves the HAPS membership by advising them on the best practices related to human body donors (also known as anatomical donors) and the laboratories they reside in. HAPS is an international organization for educators who teach anatomy and physiology across all academic levels, ranging from high school to graduate‐level health professions. The overall goal of ADS is to support members with proper and ethical utilization of human body donors within education. As such, ADS currently focuses its efforts on three main areas: laboratory resources, ethics, and coaching. For laboratory resources, ADS provides details and support for members on how to properly obtain, use, and maintain body donors, as well as provide support and feedback on anatomy laboratory design. For ethics, ADS works with members to ensure that donors are cared for ethically and responsibly and provides guidance on memorializing and honoring anatomical donors. As for coaching, ADS provides professional instruction opportunities through the ADS Support Team. The ADS Support team was formally launched in 2022 and provides support and professional development for members through one‐on‐one virtual and in‐person coaching, as well as hosting events at official HAPS conferences and workshops (e.g., institutional programs such as the University of Alabama—Birmingham Gross Anatomy for Teacher Education course). These coaching meetings and structured workshops match interested members with coaches to work on their area(s) of need, which may include general dissection skills, body region‐specific dissections, laboratory design, or even ethical questions concerning the treatment and storage of human remains. The demand for financial support to facilitate these efforts also led to the launch of the HAPS ADS Coaching Travel Award in 2025, where coaches and/or coachees can apply for funding support through HAPS to visit each other or specific workshops for hands‐on experiences.

### Anatomy educator landscape

When exploring the current landscape of anatomy educators, a recent study found that only 45% of the respondents (*n* = 654) held a PhD in anatomy and only a concerning 48% reported completing graduate coursework in anatomy and physiology.^1^ Even more alarming, only 32% reported exposure to human anatomical dissection during their anatomy teaching training.^1^ Furthermore, there are a variety of professionals teaching human anatomy across higher education in the United States, ranging from those who earned a doctoral degree in anatomy or a related field, to healthcare professionals (e.g., MD, MBBS, DPT), to interdisciplinary teaching teams (e.g., PhD, MBBS, and DPT co‐teaching), to others who do not hold a terminal degree (e.g., master's degree).^1^, ^2^, ^3^ This information, in conjunction with the decline of doctoral graduates in anatomy^4^ and the number and rate of anatomy faculty retiring,^5^ poses a concern to the future of anatomy education, especially related to human anatomical dissection. This, in turn, impacts the trajectory of health sciences education, which still commonly includes anatomical dissections and/or prosections in their curricula. Collectively, with a decrease in the number of formal anatomy doctoral programs, retirements, and various professionals teaching human anatomy, some with limited human donor interaction, there is an accelerated need for human anatomy‐focused continuing education and faculty development.

It should be noted that many of the studies described above include data primarily from faculty teaching in undergraduate and graduate/professional programs, with less data from community college faculty and high school teachers. According to HAPS membership records, as of 2025, 38% of members work at a university and 22.4% work at a college. The remaining 39.6% work at specialty programs, community colleges, high schools, or other areas. While these numbers are not composed solely of anatomists and include physiologists and other disciplines, HAPS and ADS leadership responded to member feedback and launched the Anatomy Support Team to better prepare anatomy and physiology educators to teach in these disciplines. To support this mission of growth, any HAPS member may apply for the ADS Support Team and list their coaching needs by filling out a form on the HAPS website (https://www.hapsweb.org/anatomy‐donor‐stewardship‐support‐team/). As dissection training is one of the most commonly reported needs by coachees, the ADS dissection workshops are an integral part of this program.

### Theoretical framework

The ADS Support Team was established to create working relationships between experts (coaches) and learners (coachees) using established coaching techniques and methods. Coaching techniques are individual approaches and/or skills that can be used within the coaching relationship, while models combine several techniques under one umbrella. There are several techniques that are used within coaching relationships^6^:

**Directive coaching** is authoritative and instructive, providing specific guidance, solutions, and instructions to the coachee. In this technique, the coach directs the conversation. The directive approach is particularly appropriate when the dissections must be precise, and there is not much room for flexibility.
**Nondirective, or facilitative coaching** utilizes open‐ended questioning, active listening, and allows the coachee to explore their thoughts and solutions. Here, the coach refrains from providing direct answers and encourages self‐discovery. In longer‐term coaching situations when there is time for dialogue, a nondirective application can be appropriate.
**Autocratic coaching** involves the coach making decisions on behalf of the coachee without very much collaboration. The coach provides clear instructions and expects compliance. This technique might be used when time is limited for dissection and is commonly used in prosection approaches.
**Democratic coaching** involves joint decision‐making and collaboration between the coach and coachee. The coach seeks input and considers the coachee's perspective in the decision‐making process. Dissections can be approached more than one way, and often dialogue between coaches or even small groups can result in a unique dissection highlighting important anatomical relationships.

Additionally, a number of coaching models exist to guide coaches and coachees through the skill‐learning process. A succinct description of the GROW, OSKAR, CLEAR, FUEL, and peer‐coaching models is summarized below:

GROW stands for Goals, Reality, Options, and Will^7^

- Goals—the coach helps the coachee determine their goals by setting SMART goals (specific, measurable, achievable, relevant, and time‐bound).
- Reality—coach asks questions to identify the coachee's weaknesses and challenges.
- Options—the coach and coachee brainstorm different options and strategies to close the gap between goals and reality.
- Will—coach helps the coachee draw an action plan for achieving their goals, including identifying specific steps, assigning responsibilities, and setting deadlines.
  OSKAR stands for Outcome, Scale, Know‐how, Affirmation, Action, and Review^8^
- Outcome: The coach helps the coachee define the desired result by setting SMART goals. SMART goals provide clear direction and remove distractions.
- Scaling: The coach and coachee evaluate the scale of the goal, considering its feasibility and potential impact to make sure it is realistic and aligned with the coachee's bigger plans.
- Know‐how and resources: The coach and coachee then determine the skills and resources needed to reach the goal, which include mentoring, training, or access to specific tools or equipment.
- Affirm and action: The coach assists the coachee to affirm their strengths and beliefs. They create an action plan, which outlines the steps and the deadlines.
- Review: The coach and coachee regularly review progress, identify other obstacles, and adjust to the action plan as required. This guarantees that the coachee stays on track and progresses toward the goal.
  CLEAR stands for Contract, Listen, Explore, Action and Review^7^
- Contract: The coach and coachee list the goals, expectations, and limitations of the coaching process.
- Listening: The coach uses open‐ended questions to understand the coachee's concerns, perspectives, challenges, and aspirations.
- Exploring: The coach encourages the coachee to reflect and explore underlying beliefs and patterns influencing their behavior.
- Action: The coach and coachee collaboratively develop a plan of action, outlining SMART goals, actionable steps, and necessary resources to achieve the desired outcomes.
- Review: The coach and coachee meet regularly to evaluate the progress. The coach provides feedback, celebrates achievements, and adjusts the action plan when necessary.
  FUEL stands for Frame the Conversation, Understand the Current State, Explore the Desired Goal, and Lay Out the Plan^9^
- Frame the Conversation: The coach and coachee agree on the purpose of the coaching relationship, the process, and the result.
- Understand the current state: Next, the coach asks open‐ended questions to discover the coachee's present situation and their values, strengths, and available resources. During this discovery phase, the coach also tries to identify any weaknesses and obstacles that are getting in the way of the coachee achieving their goals.
- Explore the desired goal: Envisioning their desired future state, the coach helps the coachee set SMART goals. Together, they explore options and methods for achieving the targeted outcome.
- Lay out the plan: Once the coachee decides on options and goals, they draw an action plan listing specific steps and timelines. KPIs are created to track progress. The coach regularly checks with the coachee for feedback and support.
- Peer coaching is a collaborative space where reflective practice, goal setting, and supportive feedback are exchanged between coach and coachee with the goal of developing professional growth and identity.

### The coach and coachee relationship

To initiate the coaching relationship, intentional connections were made by matching coaches and coachees who had similar regions of interest. Namely, ADS coaches draw from their experience to provide peer‐expertise that addresses the coachee's short‐ and long‐term needs; formulate goals and establish plans to accomplish those goals; identify stakeholders and pathways to meet their requirements; and establish plans for success. The ADS Support Team offers access to experts and resources in the areas of human donor ethics, integration of technology in the gross anatomy laboratory, laboratory management, scalability, sustainability, donor dissection‐related curricular reform, and faculty development. While the support offered by ADS is not limited to human dissection coaching, that is the focus of the workshop described in this paper.

Overall, ADS coaches can provide succinct, direct advice, or they may provide a short‐ or long‐term forum to guide coachees through a more complicated skill‐building process. Depending on needs and availability, the coaching process may occur asynchronously via e‐mail or synchronously via virtual meetings, or with in‐person visitation. Coaches and coachees may exchange roles when appropriate or necessary, as the contributory potential of each party reveals itself through the coaching process. Many times, the interaction between ADS coaches and coachees results in synergistic outcomes to improve the laboratory environment and/or dissection experience for both parties, which ultimately benefits students of both roles. Regardless of the technique and/or coaching model used, it is important to arrange the approach via the needs of the learner and the relationship established between the coach and coachee.

While the ADS Support Team continues with relationships and pairings between two individuals, there was a notable gap in using more team‐oriented opportunities using these coaching techniques and methods. The 2025 HAPS Pittsburgh Annual Conference dissection workshop provided the opportunity to bring multiple coaches and coaches together under a single event. The coaches in the dissection workshop were all members of the ADS Support Team and had previously forged relationships with coachees. Some coaches had a single coachee, while others potentially had multiple coachees. For coachees, some entered the workshop as non‐members of the ADS Support Team and without a coach, while others had pre‐existing relationships with a coach of the ADS Support Team. Most of these coach/coachee interactions were short‐term and utilized dissection‐specific problem‐solving techniques rather than coaching methods better utilized in long‐term coaching relationships.

Until 2025, many HAPS conferences have been hosted by institutions with only sporadic access to anatomical donors. In some cases, such as the 2018 HAPS Annual Conference hosted by Ohio State and the 2022 HAPS Midwest Dissection Workshop hosted by Briar Cliff University, human body donor dissection was only demonstrated to HAPS members or was only offered to a small number of participants located close to the host institution. These experiences were always hosted by HAPS members, but not always by the HAPS ADS and the Support Team. As such, prior to 2025, the ADS Support Team hosted the Annual Conference and only offered workshops using animal dissections (e.g., cow hearts, sheep brains) for members, with comparative anatomy and emphasized clinical correlations. This was due to previous host institutions not having human body donor labs at the conference site.

This manuscript aims to describe the development and implementation of this inaugural coaching dissection workshop. Additionally, the coaches who were involved with the Coaching Workshop (who are the authors of this manuscript) have provided reflection statements detailing their experiences, interaction with the coachees, and the impact this coaching workshop had on them over the two‐day event.

## DESCRIPTION OF THE ADS COACHING WORKSHOP AT THE HAPS 2025 ANNUAL MEETING

### Facility

The 2025 HAPS Annual Conference was located in Pittsburgh, Pennsylvania, at the University of Pittsburgh. This institution has a human anatomy laboratory for its various professional programs and access to numerous human anatomical donors. The space included standard dissection tables for all donors and common tools (bone saws, hammers, chisels, bone snips, etc.). The lab was also equipped with TVs to allow for projection of materials.

### Preparing for the dissections

ADS approached the conference co‐chairs to express interest in using their anatomy laboratory for a dissection workshop approximately a year before the 2025 HAPS Annual conference. ADS then met with the anatomy laboratory manager to discuss donor acquisition, cost, logistics, policies, and to identify additional resources available for the workshops. For the workshop, the laboratory manager approved use of donors that had not been fully dissected as part of the traditional curricula taught in the anatomy courses (i.e., medical and dental courses). The donors available had not been dissected in the head and neck and musculoskeletal regions, resulting in two workshops: “Getting ‘A‐Head’ in Dissecting: Coach‐Guided Dissections of the Head on Human Body Donors” and “Don't Be ‘Disjointed’ When Dissecting: Coach‐Guided Musculoskeletal Dissections on Human Body Donors.”

While pre‐registration for the workshops was required, interest was high. Registration was capped at 40 individuals long before the workshops took place. Each workshop was targeted to cover an anatomical region, with dissections ranging across difficulties for various levels of experience. For example, in the workshop focused on the head, dissection stations ranged from approaches to skin removal on the neck, to brain removal, to deep dissections (e.g., infratemporal fossa, tympanic cavity). Similarly, in the workshop for the musculoskeletal system, dissection stations ranged from skin removal and suturing on the back, to brachial plexus dissections to expose as proximally as the roots, to deep isolated joint dissections.

Prior to the workshop, coaches created several prosections to demonstrate the desired final product. Coaches had, on average, 12 years of experience teaching human dissection, and each coach focused on the dissection where they had the most expertise. For example, two different coaches dissected several lower‐limb joints using both superficial and deep methods. This was done to provide coachees with options depending on which anatomical relationships they wished to highlight in future dissections or based on what was feasible at their institution. Additionally, since brain removal was to be a highlight of the workshop, the coaches met the day before to remove the scalps and score some of the skulls of the donors. The purpose of this was to allow for more exploration and discussion during the actual workshop for more advanced dissectors who were comfortable with those preliminary steps. At the same time, coaches met and organized the 13 donor tables so that the “beginner” and “advanced” stations could be grouped together. Prosections were placed in the middle of the room with plenty of space around the tables so that participants would be able to see during a demonstration. Demonstrations were also aided by a number of atlas images that were projected on screens around the lab.

### Dissection day

Both workshops were facilitated by the same seven ADS coaches and each session was capped at 40 participants, with many participating in both sessions. When attendees arrived in the laboratory, they were provided with proper personal protective equipment, were questioned by coaches on their interest and level of experience, and were offered their choice of dissection station for the designated workshop. Based on the number of participants interested in each workshop, the number of donors (*n* = 13) and the coachee's level of self‐assigned experience, the facilitators divided themselves to maximize the participant‐to‐facilitator ratio. Coaches then described the different prosections to the coachees in their groups and instructed them on how to perform each dissection. Coachees worked in small groups (2–8 individuals per group) to complete their own dissections, and coaches rotated through their section to answer questions and troubleshoot. In some circumstances, coachees would identify a dissection or area of curiosity, and the coach would lead them to another donor so that they could work on a different or more directed dissection. In this way, coaches actively collaborated with coachees to address their individual goals. Additionally, the University of Pittsburgh allowed participants to explore their collection of prosections, pathologies, and anatomical variations.

## COACH IMPACT STATEMENTS

The authors of this manuscript were all coaches during the Dissection Workshop. As a moment of reflection, the coaches wrote personal impact statements describing their main takeaways and perspectives from both days of the Coaching Workshop. These statements are shown in full below with the author indicated by their initials:

**KS:**

*“As someone who has had the benefit from working in different gross anatomy labs and with a number of other anatomists, I am fairly confident in my dissection skills. Nevertheless, these dissection workshops facilitate a far greater exchange of knowledge for both novel dissectors and experienced anatomists. I have hosted smaller scale dissection workshops twice before, but the HAPS 2025 Annual Conference Dissection Workshop was the largest I have ever had the privilege to participate in. In each case, I have been amazed when I talk to my colleagues from other schools. Informal conversations with my peers reveal new dissection techniques, anatomical variations, previous unheard of lab resources, and even novel human body donor ethical considerations. These dissection workshops create the perfect space to exchange this type of valuable knowledge, not just for novel dissectors, but experienced gross anatomists*.

*During each workshop that I have led or helped lead, I have worked to plan dissections that would be meaningful to the workshop participants rather than thinking about what I would like to gain. Throughout this last workshop in Pittsburgh, I once again found great pleasure in guiding dissections and helping participants find the coach that matched their specific needs. Nevertheless, the thing that will probably stick with me the longest was something that I had not expected. I come from a racially homogenous part of the country where most people have the same color of skin as myself. During this workshop I was fortunate to work with human body donors of many skin colors. This was the first time in my whole career where I had this opportunity. Many of us have taught our students that we are all the same under our skin; however, for me, this workshop was a poignant reminder of how our humanity connects us all.”*

**BJL:**

*“On Day 1 of our dissection workshops, I assisted in the coaching of the brain removal process. We had prepared 4 body donors in different stages of the process: 1 was left completely intact, 2 were skinned and scored with an autopsy saw, and 1 was skinned but not scored. This preparation allowed the attendees to see and participate in various stages of the brain removal process. I used a donor that had been skinned and scored prior to the workshop. The scoring was only partially complete, though, so that each of the attendees in my group got to use the autopsy saw and the mallet/chisel. I described variations in methods, but since my group was done early, I took them around to see the other brain removal groups where each of the other instructors utilized different methods, so the attendees got to see the various methods of brain removal. There were also 4 donors in the lab that had their brains previously removed and skulls were bisected, which allowed for another view of the crania for attendees*.

*On Day 2 of our dissection workshops, I assisted in the coaching of dissecting/teaching the brachial plexus and lumbosacral plexus. However, once I polled the group of attendees on what they wanted to learn, I found that no coachee was interested in brachial plexus anatomy and that 1 attendee had no dissection experience, whereas the others in the group were more experienced. I taught the novice dissector how to load a blade and then how to dissect the lower limb, beginning with skinning. While she worked, I had discussions with the other attendees about various different dissections, clinical issues, and surgical procedures. Once the novice dissector got to the muscles of the thigh, I assisted her in identifying the muscles, and then I coached the group through the dissection of the knee. I then taught the group how to remove the tongue and larynx in one unit, which no one had seen before. Once we were done with that donor, some of the group went to the other area of the workshop focusing on joint dissections, and I mingled with other workshop participants pointing out various clinical issues on the donors and answering any other questions about dissections. Though this workshop did not go as planned (i.e., no one wanted to look at a plexus), it turned into a personalized coaching session where attendees could ask anything and learn what they wanted.”*

**JJG:**

*“The aspect that was the most striking from the brain removal workshop was the ability for the coachees and coaches to see how each approach aspects of dissection, including tools used, techniques, problem‐solving approaches, and teaching modalities. One of the highlights of the group I led was having faculty and students as Coachees. This led to great conversations about clinical applications, as many were focused on undergraduate A&P content. I was able to bring in my experience working in a medical/dental curriculum and discuss clinical vignettes throughout the dissection process (e.g., we identified the pterion and middle meningeal artery to discuss epidural hematomas). Once the dissection was complete, everyone was free to explore others' dissections, including the more advanced small space dissections. During this time, one of the attendees found a donor whose COD was dementia. They asked me if that could be seen on the gross brain. I explained how the brain could appear grossly and also briefly discussed pathology (e.g., Tau protein). They became slightly emotional and noted how their mother was diagnosed with dementia, and it was hard and surreal for her to see this in the lab. While talking with her, I discussed how I periodically have gotten emotional in the lab due to findings that remind me of a loved one. I mentioned how these feelings are natural and to remember that our students sometimes experience the same things*.

*For the second day, I mainly floated around helping groups as they worked on different dissections. It was great seeing the breadth of coverage in teaching we were doing as a team—from initial skinning/techniques to deep dissections. A couple of attendees asked me to show them my approach to a deeper brachial plexus dissection (i.e., to the roots). I explained how, when I prepare this, I do one side with the scalene mm. and the other side I remove them. With the scalene muscles, I pointed out the phrenic nerve. They were excited to see it, and so I asked them why the cervical level innervates the diaphragm with it being so inferior. This led into a conversation about embryology and the way you can integrate that into lab.”*

**JJW:**

*“The lab space, facilities, resources, and staff at the University of Pittsburgh School of Medicine were incredibly impressive. I can't think of a better place to do inaugural human donor dissections for HAPS. I would like to recognize everyone in the lab, especially Dr. Natasha Baker. Not only did she co‐host the conference, but she devoted extra care for our ADS Coaching team and supported our workshops to the fullest extent. None of what we did would have been possible without her*.

*I helped primarily with dissection of the head and neck small spaces on the first day and the major synovial joints on the second day. In addition to dissecting the joints, I led a brief suturing tutorial. Having the opportunity to prosect some donors the day before the workshops was key to our success, giving our team a resource of dissected examples for the participants. It also gave our team members license to show different dissection approaches*.

*Every participant whom I worked with had previous experience dissecting human donors, but did not necessarily have the time at their institutions to explore the anatomy in greater depth beyond their instructional design, or only knew techniques that they had been directly taught. The workshops provided an opportunity for participants to discuss curricular design options and to try new dissection techniques offered by our team members. I also personally enjoyed learning how my ADS Coaching colleagues designed their courses, approached their anatomy dissections, and what techniques they had developed.”*

**AB:**

*“Learning is valuable at every stage of my career, and I felt incredibly fortunate to participate in the dissection workshop at the University of Pittsburgh during the 2025 HAPS annual conference. My role in the workshop was to facilitate a brain removal demonstration for our attendees on the first day. This involved removing the scalp, cutting the cranium, and extracting the brain for further exploration. While I came into the workshop eager to share a few tips and tricks of my own, I left with a deeper appreciation for collaboration and community than I had anticipated*.

*My goal during the brain removal was to emphasize “teaching moments” for A&P faculty. For example, when making incisions on the scalp, I didn't just demonstrate how to perform the cuts, I also explained why they were made in a certain way and discussed the superficial blood vessels and nerves at risk. When cutting through the cranium, I highlighted anatomical landmarks that could guide cleaner access and preserve brain integrity. These moments naturally prompted questions and insights from the attendees, which created space for others to share their own techniques, some of which I hope to incorporate into my own teaching. It truly became a collaborative effort, and watching participants gain confidence as they made incisions and bone cuts was an experience I'll never forget*.

*One moment that stands out occurred after the dissections were complete. A participant approached me and asked, “How do you know all of this? What textbooks or videos did you use?” I paused, realizing that I hadn't relied on those resources. My knowledge came from doing exactly what they were doing, observing experienced faculty, working closely with mentors, staying late in the lab to practice dissections, and learning by doing. It made me realize that not everyone has had the privilege of access to donors and hands‐on opportunities, and that reinforces the critical role ADS plays in supporting HAPS members through workshops like the one in Pittsburgh*.

*The gratitude from attendees was energizing. I left the workshop with new clinical correlations that I hadn't considered before, as well as future opportunities to collaborate and share resources. The sense of community and the rich educational exchange fostered a spark of professional growth that I hope to carry forward!”*

**DNE:**

*“Opportunities for anatomy educators to gain experience and knowledge from colleagues are significantly limited. I have hosted dissection workshops for anatomy educators in the past and so I was extremely excited for the opportunity to continue this at a conference because I know the benefits and opportunities they award to both the attendees (the coachees) and the preceptors (coaches)*.

*Most of the coachees I interacted with were more on the more novice side in dissection with both faculty and students. It was amazing to see that several faculty brought some of their students who were interested in anatomy education as a career to the workshop in order to gain more experience. Overall, I ranged from instructing on how to use instruments, similar to first day of lab in my own classrooms, to showing and discussing different approaches to some of the deeper spaces in the head and neck region. They had a theoretical understanding of the space through coursework but had not been able to explore it in a dissected space. This meant tool safety (bone saws in particular), approaches, and considerations all were needed to be discussed. This was a completely different experience on my end from instructing students, as the Coachees already had a good grasp on the anatomy but getting that hands‐on experience and seeing the actual size, depth, and organization, as well as all the variants we always run across, brought out a different set of emotions from awe, to amazement and surprise. Consistent exclamations of “I didn't realize they were so small” particularly to nerves and “Why is it doing that” to variations were common. Additionally, being able to discuss the trial‐and‐error process and how a person's anatomy can change the approach is not something you generally think about, but can be helpful when learning the anatomy*.

*An additional thing that I was not expecting was how much the coaches, including myself, were able to learn from each other. I do not have a ton of experience in joints, but I was able to see the approach and different structures that I generally have only learned from coursework. At the end of the day, several of the coaches practiced ear dissection with hammer and chisel as this was not as commonly done at their schools. Collectively, I think the coaches were as impacted by the event as the coachees were*.

*Throughout the two‐day workshop, both the coaches and coachees all had very positive things to say about the experience. After the workshops ended, the conversations didn't. Particularly, coachees often spoke of how they were going to take their newfound knowledge back to their classrooms. The best thing to hear as everything was wrapping up was “Are you going to do another one next year?”*

**RER:**

*“Instruction in the anatomy lab is my passion. I approach dissection with the knowledge that the tactile interaction, intimacy, and naked honesty of it leaves an indelible impression on students and faculty alike. Opportunities to share that with students or colleagues outside of a traditional laboratory setting are very few and far between, and often costly to some extent. Opportunities to even do that within one's own school on an elective basis is also a pretty rare opportunity*.

*My role at the workshop was to walk truly novice dissectors through their initial dissection experience, first on the head with the end goal of a calvarium removal on the first day, and similar novice‐level instruction for limbs the following day. I took several dissectors under my wing to introduce them to an embalmed human anatomy donor, considerations to take that you might not think about when working with an animal (e.g., greater degree of dignity, privacy, etc.), reminding them of the layers of the body and describing how interacting with each of these layers will be as different as each tissue layer is structurally different. They were shown how to load a blade onto a scalpel, how to pass instruments with proper surgical technique, how to consider and describe homologous structures between human donors and animal specimens, how to close incisions, and how to dissect their donor utilizing the more loosely held spaces between tissue planes to prevent undue trauma to the body*.

*These colleagues all had an excellent grasp of the anatomy at hand—they taught it, obviously, but mostly only with animals or with textbook knowledge. They had no real interaction with human anatomy in humans—and thus gained a real appreciation for the composition, organization, and inherent variation present from donor to donor*.

*This appreciation and the awe for the experience that came with it were what I was so thrilled to see them take home. This appreciation translated to excitement for next year's conference and their next chance to hone their newfound skills—every person that I worked with at those workshops asked me what next year's dissection workshop would be!”*

## SUMMARY OF REFLECTIONS

Overall, a number of common threads emerged upon reflecting on the HAPS ADS Pittsburgh dissection workshop. Collaboration between coaches and coachees was experienced universally. Additionally, it was clear that the coaches were flexible and open to the needs of the coachees. This seemed to be the primary objective for coaches adapting dissections to meet the individual interests and skill levels of the workshop participants.

## DISCUSSION

Overall, the dissection coaching workshop demonstrated the value of hands‐on experiences that provide professional development opportunities for anatomy educators across varied institutions. A key outcome of the workshop was the exchange of knowledge between novice and experienced anatomists. Rather than functioning as a hierarchical training model, the workshop fostered a collaborative learning environment where participants openly shared dissection techniques, teaching strategies, and curricular approaches. Coaches noted that they learned from each other as well as from the individuals they guided, reinforcing the workshop as an active professional learning network.

The protocol used to match coaches to coachees, as well as the logical application of these various coaching models, has been outlined in (Figure 1).

**FIGURE 1:**
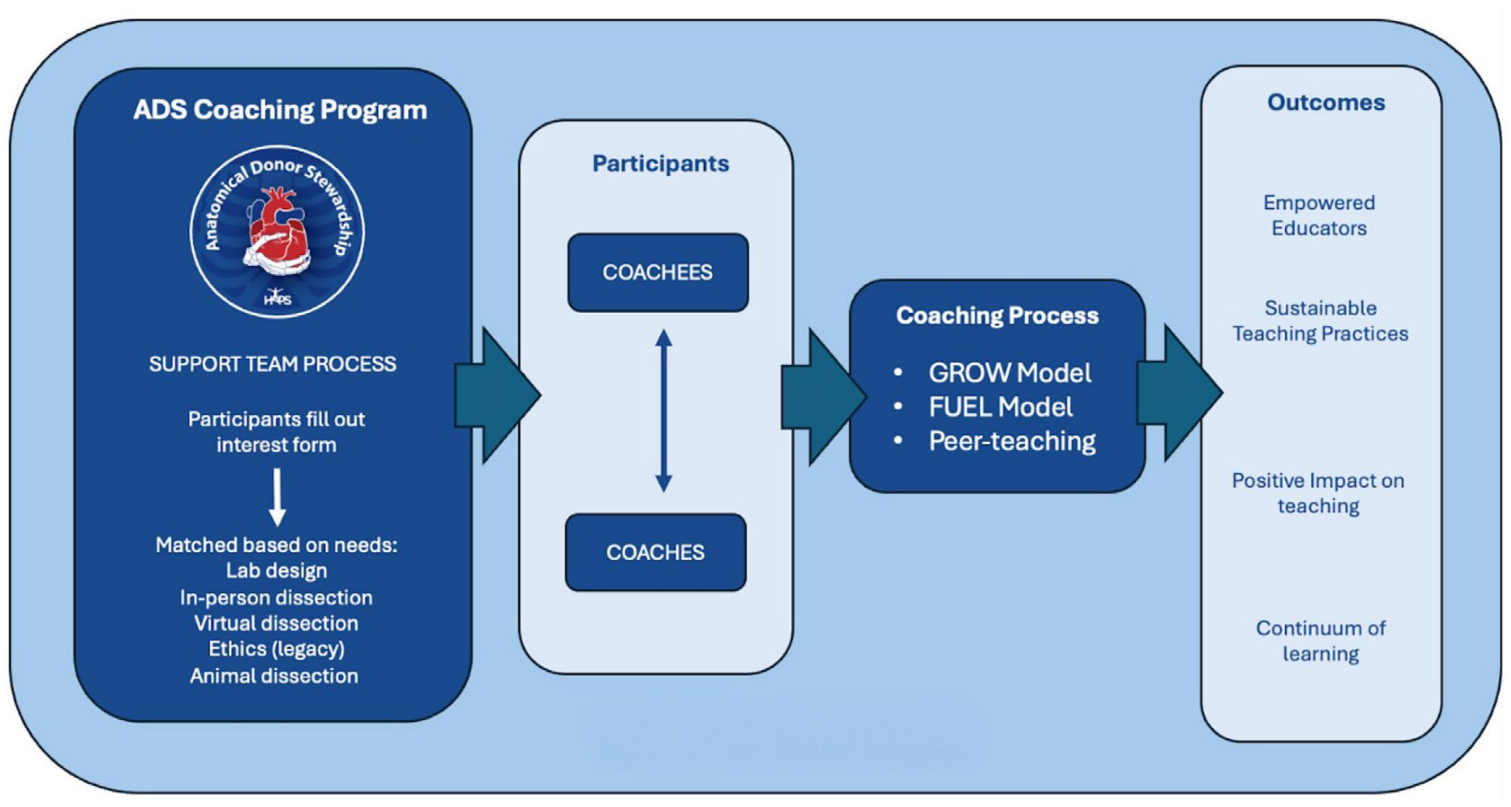
Diagram showing the coaching model used by the HAPS ADS Support Team.

Within the dissection workshop, both Nondirective and Democratic coaching techniques were employed in a flexible, hybridized peer‐coaching model that facilitated tailormade problem solving and dialogue between each coach and coachee pair or small group. These techniques provided coaches with the ability to provide direction and structure to dissections, but also allowed coachees to explore anatomical regions, attempt dissections, and learn at their own pace and experience level. The following describes an explanation of how the GROW, FUEL, and CLEAR coaching models were blended in the Pittsburgh workshop:

The GROW model was primarily used for its collaborative and intentional coaching strategy. This was accomplished in the workshop as coaches were able to help identify coachee needs and help them tackle their dissection needs face to face in the University of Pittsburgh lab. These collaborations were conducted both one‐on‐one and in small groups. Since part of the GROW method is to identify challenges, coaches were also able to use the workshop collaborations to describe common complications associated with each dissection as well as possible solutions. This typically involved facilitative or democratic coaching where the coachee was able to ask questions and propose their own ideas. The FUEL model was utilized in situations where peer teaching was explored between coaches and participants. Often participants were able to step in and help provide direction to their peers. This also occurred between coaches. Last and less frequently, the CLEAR coaching method, which is also collaborative, was used for longer‐term coaching relationships to discuss goals and make longer‐term plans for future remote and/or in‐person coaching interactions.

A major strength of the workshop was its responsiveness to participants' individual needs. Coaches were able to adjust their instructional focus based on participants' skill levels, interests, and prior experience. In several instances, planned content shifted organically when participants expressed different learning priorities, leading to more meaningful engagement. This flexibility reflects a learner‐centered approach that is especially valuable in dissection‐based education, where participants often arrive with varied exposure to human donor work.

The workshop also provided rare access to human donors, a resource that is not consistently available across institutions and is rarely offered to outsiders when it is available. This hands‐on dissection experience deepened anatomical understanding and strengthened professional identity for many participants, particularly for those accustomed to teaching primarily from models, digital platforms, or non‐human specimens. Direct interaction with human donors allowed all participants to appreciate anatomical variation, tissue properties, differences in comparative anatomy, and spatial relationships in ways that cannot be fully conveyed through theoretical or simulated anatomy.

In addition to technical development, the workshop created space for emotional and humanistic engagement. Encounters with donors prompted reflection on empathy, dignity, and the lived experiences of the individuals who gifted their bodies. Some participants expressed emotional responses when encountering conditions that connected to their personal lives, highlighting the importance of acknowledging affective dimensions of anatomy education. These experiences underscore the role of our anatomy laboratories in shaping not only cognitive knowledge, but also professional identity and compassionate practice with our students, both aspects of the “hidden curriculum.”

While the workshop was highly impactful, several areas for improvement emerged. A structured pre‐workshop needs assessment, such as a survey asking participants what area(s) they would like coaching on, could help align prepared activities with participant goals from the outset. Additionally, incorporating intentional reflective debriefing sessions would set aside time for participants to process emotional responses and consider how to support similar experiences among their own students. Finally, sustaining the momentum and nurturing the community created during the event remains a challenge; establishing ongoing resource‐sharing networks or follow‐up programming may help maintain engagement.

Overall, these findings suggest that human donor coaching workshops can serve as powerful and equitable professional development experiences. By emphasizing collaboration, providing access to donors, offering reflective practice, and curating flexible instructional approaches, the model has strong potential to enhance anatomy education across diverse institutional settings.

### Key takeaways

- *Collaborative, Mutual Learning*: The workshop supported knowledge exchange among novice and experienced anatomists, rather than one‐way instruction.
- *Learner‐Centered Coaching*: Coaches adapted dissection activities to participant needs and interests, demonstrating flexible and responsive teaching and learning.
- *Expanded Access to Donor Dissection Experience*: Participants gained rare hands‐on donor experience, deepening understanding of anatomical variation and dissection technique.
- *Integration of Clinical and Pedagogical Strategies*: Dissections were paired with teaching tips, clinical connections, and laboratory management insights transferable to participants' home institutions.
- *Attention to Humanistic and Emotional Dimensions*: The workshop acknowledged the emotional weight of working with donors, reinforcing reflection, respect, and empathy.
- *Future Improvement Opportunities*: Structured needs assessments and ongoing post‐workshop community support would further enhance the program's impact.

### Future directions

In this reflection, it was noted that the University of Pittsburgh was incredibly supportive and responsive to our needs and led to the success of the workshop. It is paramount that future workshops have the same support and response from the host institution as well as the host society administration in order to scale the program at a national or international level. There is a significant need for extensive administrative support to develop, host, and provide similar coaching workshops for members within and outside the society.

As a pilot, this study lacked some additional information. First, this study did not collect formal feedback from the attendees in the form of a survey. This lack of the lack of survey for the needs of members was a noted weakness. This survey could include questions relating to geographic regions to guide locations of future workshops, anatomical regions of interest, and what an individual is willing to pay for this type of workshop. In addition, post‐workshop surveys gauge their thoughts and perceptions of the sessions, including strengths and areas for improvement that are needed. Another consideration is that even with the feedback and suggestions for future workshops, the logistics of available resources (e.g., donors, facilities) at different conferences will differ, and the ability to match them to the needs of participants may not always be possible. Ultimately, the ADS will need to make decisions about how to meet the needs of as many of the participants as possible. This task can be managed by working with a diverse committee to design future workshops and using additional resources such as sister organizations as a means for a more global reach.

Additionally, the coaching workshop is limited in access and scope. At present, the coaching workshops are currently hosted as part of an annual conference, but this is likely not sufficient to meet the needs of current members, does not help members who cannot attend conferences, or those who are outside of membership altogether. Additional workshops at regional conferences or even standalone events at academic institutions can help diversify the workshops while meeting the most needs. Not only can this increase location access, but it can also decrease the financial burden.

## CONCLUSION

The use of coaching models for human dissection skill development had not been previously used before this workshop. Based on the development of this workshop and the coaches' reflections, additional coaching workshops could be of benefit to members. The potential reach of this model could extend to other HAPS initiatives; for example, other HAPS committees may seek to foster other coaching and mentorship relationships that support teaching skills and scholarship.

## AUTHOR CONTRIBUTIONS

**Jonathan J. Wisco:** Conceptualization; writing – review and editing; writing – original draft. **Kelsey E. Stevens:** Conceptualization; writing – original draft; writing – review and editing. **Jeremy J. Grachan:** Conceptualization; writing – original draft; writing – review and editing. **Abbey Breckling:** Conceptualization; writing – original draft; writing – review and editing; visualization. **Bobbie J. Leeper:** Conceptualization; writing – original draft; writing – review and editing. **Danielle N. Edwards:** Conceptualization; writing – original draft; writing – review and editing. **Rhiannon E. Robinson:** Conceptualization; writing – original draft; writing – review and editing.

## CONFLICT OF INTEREST STATEMENT

The authors have no conflict of interest to report.

## ETHICS STATEMENT

Ethical approval was not applicable for this study.

## Data Availability

Data sharing not applicable to this article as no datasets were generated or analysed during the current study.
